# Gender disparities in clinical practice: are there any solutions? Scoping review of interventions to overcome or reduce gender bias in clinical practice

**DOI:** 10.1186/s12939-020-01283-4

**Published:** 2020-09-22

**Authors:** Lorena Alcalde-Rubio, Ildefonso Hernández-Aguado, Lucy Anne Parker, Eduardo Bueno-Vergara, Elisa Chilet-Rosell

**Affiliations:** 1 Department of Public Health, History of Science and Gynaecology, School of Medicine, University Miguel Hernández, Crta Nacional, N-332, s/n, 03550 Sant Joan d’Alacant, Spain; 2 CIBER de Epidemiología y Salud Pública (CIBERESP), Madrid, Spain

## Introduction

Gender, understood as “social relationships between males and females in terms of their roles, behaviours, activities, attributes and opportunities, and which are based on different levels of power”, [1] is one of the main social determinants of health [2]. The damage caused to population health by gender inequality across the globe is immense and justifies comprehensive actions addressing gender equity in health at all levels [3]. In the words of Hawkes and Buse, “Now is the time to take the call from Alma Ata in its literal sense—“Health is for All” not only for some. Embedding of gender in global health provides one promising route to attainment of the longstanding, but long-languishing, human right—the right to health” [4]. The root causes of gender inequality encompass all societal spheres and a multisectoral approach is required [5]. In fact, it has been shown that actions across multiple sectors in low and middle-income countries can improve a variety of health and development outcomes [6]. Therefore, there is no doubt that gender mainstreaming should pervade all policies. The UN Economic and Social Council embraced this approach in 1997 as “assessing the implications for women and men of any planned action, including legislation, policies, or programmes … so that women and men benefit equally, and inequality is not perpetuated” [7]. On global level, the impact of gender inequality on health was later included in the UN’s the Millennium Development Goals, and remains significant in the Sustainable Development Goals [8].

In the health domain, there has been a substantial interest in gender issues in the last two decades. Vlassof and García Montero explained why gender is key to understanding all dimensions of health including healthcare, health seeking behaviour and health status. Consequently, they proposed transformation in all areas of the health sector in order to integrate gender perspective [9]. This integral change should encompass actions on policy, research, training and programmes including interventions at the individual level. We have witnessed an appreciable increase in the consideration of gender in health plans [5, 10] and particularly in those focused on women’s reproductive health [11, 12]. However, more than 20 years of research from high-income, middle income and low-income countries shows that gender inequalities remain embedded in health systems [13, 14]. Within health care systems, unconscious gender biases –based on gender stereotypes- and sexism affect patient care [15, 16]. While policy and organisational changes are essential, the involvement of health workers can act as a catalyst of integral change in the healthcare system.

Since the recognition of gender bias in the clinical management of cardiovascular disease, [17–19] several other health problems have been the target of research, which shows the extent of gender inequity in health care. Last year, Nature Communications published a study analysing health data for almost 7 million men and women in the Danish healthcare system over a 21-year period, and showing that women were diagnosed later than men in more than 700 diseases [20]. Despite demonstrated disparities in women’s health and advocacy to improve women’s health, there is still a lack of patient centred care for women.

These contributions from research on the relevance of gender inequalities in health care have not gone along with research on effective interventions that could provide health workers with practical tools that facilitate the application of gender oriented clinical interventions. In addition, the lack of patient centred care for women has been reported recently [21–23]. In fact, Celik et al.’s 2010 review of the available literature, [24] the authors failed to find references that contributed to the development of procedures to increase health professionals’ skills related to gender. Health systems and health providers remain largely gender unresponsive [13]. In order to move forward we need to assess the available experience in reducing gender-based inequities and, where possible, learn how to scale-up effective interventions. Our objective here is to identify available tools that can be used to overcome or reduce gender bias in clinical practice.

## Material and methods

This scoping review was developed following the Arksey and O’Malley’s methodological framework, which we used to guide our reporting where possible [25]. We specifically searched for articles examining interventions to reduce or prevent gender bias in clinical practice, as long as they were provider-focused and healthcare-based.

### Search strategy

The primary search was performed in Medline through PubMed, Web of Science, Scielo and Lilacs. Modifications on our search strategy in Medline through PubMed were made several times to ensure highest sensitivity. Finally, we decided to combine two individual searches to expand our search in Pubmed and we then made minor modifications to adequate the search strategy to each database. The final search strategies combined Subject headings and MeSH terms related to “gender”, “healthcare”, “bias”, “disparities”,“inequality”,“inequity” and “intervention” (Table 1).

**Table 1:** Search strategies applied in each database in order to identify publications examining interventions to reduce or prevent gender bias in clinical practice

Medline through Pubmed	A. ((bias [Title/Abstract] OR disparities [Title/Abstract] OR inequality [Title/Abstract] OR inequalities [Title/Abstract] OR inequity [Title/Abstract] OR inequities [Title/Abstract])) AND (intervention*[Title/Abstract] OR reduction [Title/Abstract]) AND healthcare AND gender
	B. (gender [Title] OR “gender bias” [Title/Abstract] OR “Sexism”[Mesh] OR sexism [Title/Abstract] OR “Sex Factors”[Mesh] OR “Sex Factors” [Title/Abstract] OR “sex disparities” [Title/Abstract] OR “sex disparity” [Title/Abstract] OR “sex based” [Title/Abstract] OR “gender-based”[Title/Abstract]) AND (intervention [Title/Abstract] OR reduction [Title/Abstract]) AND (“health care” [Title/Abstract] OR healthcare [Title/Abstract] OR “health services”[Title/Abstract])
Scielo	((((ti:(gender))) OR (“gender bias” OR sexism OR “Sex Factors” OR “sex disparities” OR “sex disparity” OR “sex based” OR “gender-based”)) AND (intervention OR reduction)) AND (“health care” OR healthcare OR “health services”)
Web of science	(TS = (“health care” OR healthcare OR “health services”)) AND (TS = (intervention OR reduction)) AND (TS = (“gender bias” OR sexism OR “Sex Factors” OR “sex disparities” OR “sex disparity”)) AND TI = (gender)
Lilacs	gender [Palabras del título] OR “gender bias” OR sexism OR “Sex Factors” OR “sex disparities” OR “sex disparity” [Palabras] AND intervention OR reduction [Palabras] AND “health care” OR healthcare OR “health services” [Palabras]

In order to retrieve as many interventions studies as possible, we applied no date limitations and retrieved all results published until December 2018.

### Study selection

We included empirical studies designed to prevent or decrease gender bias in clinical practice and those that were focused on other types of prejudice (such as race, age …) as long as they also evaluated gender bias. Similarly, we included studies designed to evaluate the effect on gender bias of interventions already implemented for a different primary objective (e.g. improving adherence to guidelines). These interventions should be provider-focused and healthcare-based. We only included studies that evaluated the interventions. Given the heterogeneity in the evaluation of gender bias, we included studies that assessed or measured any outcome related to clinical practice in a gender-disaggregated way (e.g. in-hospital adverse events) or the effects of interventions designed to reduce gender-based vulnerability of specific population (LGBTI+ populations, women suffering from intimate partner violence). We only included studies that were published in peer-review journals in English, Spanish and Portuguese. Exclusion criteria included non-empirical or descriptive studies, interventions focused only on patients and description of programmes or interventions without an evaluation of the impact.

**Fig. 1:**
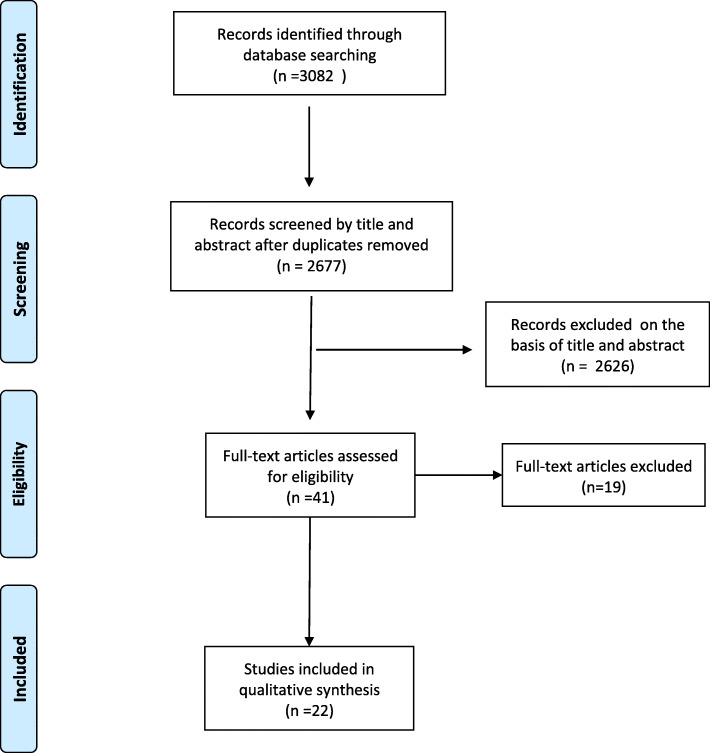
Flow diagram for identification of interventions to reduce gender bias in clinical practice

All search results were first screened based on title and abstract by two researchers. The full text of potentially useful records was reviewed. We read all potentially useful texts and their reference lists were also revised for additional interventions. A detailed flow diagram of study selection is showed in Fig. 1.

### Data extraction and synthesis

We carried out the data extraction using a standardized data extraction form. Data were collected on the health issue, country, description of intervention (later categorized in clinical decision support guidelines and standardized protocols; interventions that included staff, clinic and community interventions; interventions managed by an all women team for female patients; gender sensitive improvements in data collection, and routine screening for gender violence), type of evaluation (considering the comparison group and the use of routine or non-routine-data) clinical setting (hospital, specialized care, primary health care, and others), main results and conclusions (later classified as successful or partially successful and not successful).

In order to evaluate the application of gender perspective in research reporting, we used the SAGER guidelines checklist adapted to our data extraction form [26]. In this case, we obtained information from the following items: introduction (explanation on whether sex and/or gender differences may be expected); methods (explanation on how sex and gender were taken into account in the design of the study, whether they ensured adequate representation of males and females, and justification of the reasons for any exclusion of males or females); results (in addition to sex-disaggregated data, it includes variables that facilitate gender analysis); and, discussion (implications of sex and gender on the study results and discussion of the implications of the results stratified by sex or from gender perspective).

Firstly, we performed an initial analysis of five papers by two researchers in order to homogenize data coding. Researchers agreed in four papers. After consensus on the assessment of the main variables, we proceed with the remaining articles. For the second set of articles, two researchers extracted data independently. A third research was in charge of detect discrepancies between researchers. Discrepancies were detected in four papers and were solved by consensus between the two researchers that reviewed each paper. Those discrepancies were related to minor variations on the length of text extracted to justify their answers and did not influence the interpretation of the results.

We performed a descriptive analysis of the information obtained from items formerly described.

## Results

After removing duplicates, we screened 3082 abstracts retrieved through database search. Additional file 1: Appendix 1 presents detailed information of the 22 [27–48] studies included in our scoping review.

When reporting the interventions, information regarding sex differences and the gender perspective: two of the studies failed to include whether sex and/or gender may be an important variant of the health outcome assessed in the introduction section (9%), three of the studies failed to report how the researchers ensured adequate representation of males and females in the sample (14%), in nine of them lacked variables/information that enabled a gender-based analysis (40%). Five studies did not discuss sex differences or apply a gender perspective (23%) and six did not discuss the implications of the results from a gender perspective (27%) (Table 2).

**Table 2:** Compliance with the adapted SAGER guidelines checklist of the 22 analysed interventions aimed at reduce gender bias in clinical practice

SAGER	Recomendation	N (%)	References
	Authors reported whether sex and/or gender differences may be expected in introduction	2 (9)	[40, 43]
	Authors ensured adequate representation of males and females in methods sections	3 (14)	[44, 45, 47]
	Methods section included variables/information that enabled a gender-based analysis	9 (40)	[27, 32, 34, 36, 39, 40, 44, 46, 47]
	Authors discussed sex differences or apply a gender perspective in discussion	5 (23)	[31, 38, 40, 44, 47]
	Authors discussed the implications of the results from a gender perspective.	6 (27)	[31, 32, 34, 42, 43, 47]

The interventions analysed were mainly focused on cardiovascular disease (*n* = 13, 59%) and, sexual and reproductive health, including one intervention focused on sexual orientation and gender identity (*n* = 5, 23%). Other themes were gender-based violence (*n* = 1), unhealthy drinking (n = 1), diabetes (n = 1) and renal failure (n = 1) (Table 3). Seventeen studies were conducted in USA (77%); the others were located in Brazil (*n* = 2), India (n = 1), Tanzania (n = 1) and Singapore (1).

**Table 3:** Description of main characteristics of the 22 analysed interventions aimed at reduce gender bias in clinical practice

	N (%)	References
**Health issue**		
Cardiovascular health	13 (59)	[24, 25, 29, 31, 33–35, 37–40, 42, 43]
Sexual and reproductive health	5 (23)	[26–28, 30, 32]
Gender based violence	1 (5)	[36]
Unhealthy drinking	1 (5)	[45]
Diabetes	1 (5)	[44]
Renal failure	1 (5)	[41]
**Type of intervention**		
Clinical decision support decision support guidelines and standardized protocols	15 (68)	[24, 25, 29, 31, 33–35, 37, 38, 40–45]
Interventions that included staff, clinic, and community interventions	3 (14)	[27, 28, 30]
Interventions managed by an all women team for female patients	1 (5)	[39]
Gender sensitive improvements in data collection	2 (9)	[26, 32]
Routine screening for gender violence	1 (5)	[36]
**Type of evaluation**		
Evaluation without comparison group, routine data.	7 (32)	[24, 25, 29, 38, 40, 41, 43]
Evaluation with pre and post comparison and routine data	6 (27)	[31, 33–35, 37, 42]
Evaluation with randomised control and non-routine quantitative data	2 (9)	[39, 44]
Evaluation with a non-randomised group, non-routine mixed data	2 (9)	[28, 32]
Evaluation without comparison group and non-routine mixed data	2 (9)	[26, 36]
Evaluation without comparison group and non-routine qualitative data	1 (5)	[30]
Evaluation with pre and post comparison group, and non-routine quantitative data	1 (5)	[45]
Evaluation with pre and post comparison and non-routine qualitative data	1 (5)	[27]
**Clinical setting**		
Hospital	14 (64)	[24, 25, 29, 31–38, 40, 42, 43]
Specialized care	4 (18)	[28, 39, 41, 45]
Primary health care	2 (9)	[27, 44]
Others	2 (9)	[26, 30]
**Results**		
Successful or partially successful	19 (86)	[24–32, 34–39, 41–44]
Not successful	3 (14)	[33, 40, 45]

Interventions were conducted more frequently in hospitals (*n* = 14, 64%). We found several types of intervention. Most studies included clinical decision support decision support guidelines and standardized protocols (15, 68%). These studies were aimed to reduce variability in healthcare and were not specifically designed to reduce gender bias. There was a cluster of studies (5) related to the program in the United States called Get with the Guidelines (GWTG) [49]. This initiative was focused on the redesign of hospital systems in order to improve the quality of patients care and was based on a collaborative model and Internet-based Patient Management. The GWTG included interactive learning sessions, teleconferences, and electronic communication between multidisciplinary teams from hospitals in a variety of settings to facilitate the transfer of the “how-to”, which is considered necessary to produce system-wide change. Finally, there were interventions that included activities involving staff, clinic, and community interventions (3, 14%), two studies evaluated data collection in a more gender-sensitive way, one more was an intervention managed by an all women team for female patients (2, 9%), and finally one study evaluated the implementation of gender violence screening.

The evaluations of the interventions were mostly conducted without comparison group and using routine data (7, 32%) or with a pre and post comparison and using routine data (6, 27%). The other 9 studies used non routine data (quantitative, qualitative and mixed data) and a variety of designs: randomised control group (2, 9%), non- randomised control group (2, 9%), without comparison group (3, 14%) and pre-post comparison (2, 9%).

The majority of the interventions (19, 86%) were mostly successful in narrowing the gender gap (See Annex 1 for more details). Four of them were unable to narrow this gap in all outcomes. There was no difference in cardiovascular events, quality of life, knowledge, attitudes and practices in women with cardiovascular disease after intervention [42]. A discharge tool was less used in women after acute myocardial infarction than in men [38].. Fewer women than men with heart failure received hospital discharge instructions and the length of the stay was longer for women even after implementation [32]. Additionally, the gender violence screening raised doubts in clinicians [39]. On the other hand, three studies were not successful in any outcome: two interventions in cardiovascular disease [36, 43] and one in unhealthy drinking [48]. The latter one stated that a non-gender-specific threshold for an intervention in alcohol misuse was detrimental as may increase gender differences in receipt of brief intervention among patients.

## Discussion

Despite the extensive and growing evidence of gender bias in clinical practice published in scientific journals since the 90s, our scoping review has shown that few studies have tried to tackle this bias. After screening over 3082 abstracts in health sciences databases, we identified only 22 evaluated provider-focused and healthcare-based interventions. Most of the analysed studies focused on cardiovascular diseases and were strategies to improve adherence to existing guidelines in order to reduce variability in healthcare. It is noteworthy that even though the studies included in our scoping review described interventions that could reduce gender bias in clinical practice, we identified shortcomings in the reporting of the information from a gender perspective. Most of the interventions were successful in narrowing the gender gap in at least one of the outcomes even when they were not intended or seeking to reduce the gender gaps. Therefore, it is likely that future innovative interventions designed according to the theoretical bases that originate gender bias could result in higher reductions on gender bias.

There are, however, some limitations in our study. Firstly, the difficulty to find suitable articles, which we addressed by redefining our search and inclusion criteria several times in order to increase sensitivity. Secondly, the methodology of the studies was heterogeneous and could hinder the comparisons between studies. In addition, considering that some of the results of the analysed interventions were based on studies lacking a comparison group, interpretations should be cautious. Finally, interventions were conducted on few countries, which could difficult to replicate them in different contexts.

Although we identified few studies which sought to reduce gender bias in clinical practice, the interventions examined were mostly successful, demonstrating that narrowing gender gaps in healthcare is possible. This scoping review is a starting point, which, along with barriers and facilitators of interventions to reduce gender gap in healthcare already described in literature [24], can guide future interventions. The analysed interventions showed that gender disparities in healthcare could be reduced and even eliminated if clinician’s adherence to guidelines increased. Most of these interventions proposed the protocolization of technical procedures that aimed to reduce differences by sex and other variables without seeking specifically to reduce gender biases in health care - and may or may not result in that reduction. In contrast, interventions designed with the aim of reducing gender bias included different strategies (like programs managed by an all women team or improvement of the data collection system) and all of them were successful or partially successful in their objectives.

Most of the studies, particularly those focused on technical procedures, were based on specialized health care and hospitals. There is a lack of studies addressing this problem in primary healthcare (only two studies were based on this setting). If the narrowing of gender biases occurs in primary care, its impact could be even greater due to the volume of patients treated in these centres and because it is the patient’s first contact with the healthcare system [50].

Gender bias in clinical practice was described for the first time in the New England Journal of Medicine [17–19]. Almost 30 ago, Bernadine Healy used the term “Yentl syndrome” equating women with myocardial infarction to the character Yentl - a Jewish woman who dressed herself as a man to be able to study the sacred texts [18]. Healy was denouncing the fact that women have to show the same symptoms as men to receive the appropriate diagnosis and treatments, because the knowledge of cardiovascular disease was based on studies conducted on men. Since then, many studies have addressed gender bias in clinical practice, particularly in cardiovascular disease. In concordance with this, cardiovascular health was the predominant issue addressed in the analysed interventions. However, gender bias has been described in the clinical practice of a great number of diseases, [20] so it is necessary to expand the field of work to other health issues.

Importantly, physicians –and, the health system in general– have the potential to either reproduce or perpetuate disparities, or to overcome them. Even if the results of the interventions are encouraging, we need to question the theoretical framework in which these gender inequities originated. This may be why some interventions were not successful, as simply implementing instruments, while necessary, is not enough to tackle gender bias in professionals. It is important to advocate for reforms aimed to include gender aspects in the curricula of medical schools and in health research in order to advance in the field of gender- specific medicine [51].

## Conclusions

In contrast to the wide research identifying gender bias in health care, few studies, so far, have described and evaluated interventions aimed to tackle this bias. However, there is some empirical evidence showing how to narrow the gender gaps in healthcare, as the reviewed literature reveals that that most of the interventions were successful at achieving at least one of the expected outcomes. Nevertheless, it is alarming that studies of interventions in primary healthcare, where the impact of narrowing of gender bias could be greater, are almost absent in the present available research.

Based on the results of our review, we consider that knowledge about the causes of gender inequities in healthcare should permeate new research on how to increase gender equity and improve quality in clinical practice.

### Implications for practice and/or policy

Future clinical practice interventions should be developed with a gender perspective and should be comprehensive, long-term, experimental, evaluated with standardized methods, and specifically developed to tackle gender bias. In addition, they should address not only the women-man dichotomy, but also the gender continuum. Interventions should consider facilitators and barriers to include gender perspective in healthcare and they should always be adapted to the specific context, moment and population targeted. Finally, successful implementation is not enough, monitoring is essential. Standardized indicators and audits need to be developed for a structural embedding of gender in clinical practice.

## Supplementary information

**Additional file 1.** Appendix 1.

## Data Availability

All available data is included in the publication.

## References

[1] Manandhar M, Hawkes S, Buse K, Nosrati E, Magar V (2018). Gender, health and the 2030 agenda for sustainable development. Bull World Health Organ.

[2] Marmot M (2005). Social determinants of health inequalities. Lancet.

[3] Sen G, Östlin P, George A (2007). Unequal, Unfair, Ineffective and Inefficient. Gender Inequity in Health: Why it exists and how we can change it. Final report of the Women and Gender Equity Knowledge Network (WGEKN).

[4] Hawkes S, Buse K (2013). Gender and global health: evidence, policy, and inconvenient truths. Lancet.

[5] Östlin P, Eckermann E, Mishra US, Nkowane M, Wallstam E (2006). Gender and health promotion: a multisectoral policy approach. Health Promot Int.

[6] Taukobong H, Kincaid M, Levy JK, Bloom S, Platt J, Henry S, Darmstadt G (2016). Does addressing gender inequalities and empowering women and girls improve health and development programme outcomes?. Health Policy Plan.

[7] United Nations General Assembly (1997). Report of the economic and social council for 1997.

[8] Gupta GR, Oomman N, Grown C, Conn K, Hawkes S, Shawar YR, Shiffman J, Buse K, Mehra R, Bah CA (2019). Gender equality and gender norms: framing the opportunities for health. Lancet.

[9] Vlassoff C, Garcia Moreno C (2002). Placing gender at the Centre of health programming: challenges and limitations. Soc Sci Med.

[10] Wilkins D, Payne S, Granville G, Branney P (2008). The Gender and Access to Health Services Study. Final Report.

[11] Briones-Vozmediano E, Vives-Cases C, Peiró-Pérez R (2012). Gender sensitivity in national health plans in Latin America and the European Union. Health Policy.

[12] Boender C, Santana D, Santillan D, Hardee K, Greene ME, Schuler S. The “So What?” report: A look at whether integrating a gender focus into programs makes a difference to outcomes. Washington, DC: Population Reference Bureau for the Interagency Gender Working Group; 2004.

[13] Hay K, McDougal L, Percival V, Henry S, Klugman J, Wurie H, Raven J, Shabalala F, Fielding-Miller R, Dey A (2019). Disrupting gender norms in health systems: making the case for change. Lancet.

[14] Morgan R, George A, Ssali S, Hawkins K, Molyneux S, Theobald S (2016). How to do (or not to do)... gender analysis in health systems research. Health Policy Plan.

[15] Hoffmann D, Tarzian A (2001). The girl who cried pain: a bias against women in the treatment of pain. J Law Med Ethics.

[16] Travis CB, Howerton DM, Szymanski DM (2012). Risk, uncertainty, and gender stereotypes in healthcare decisions. Women Ther.

[17] Ayanian JZ, Epstein AM (1991). Differences in the use of procedures between women and men hospitalized for coronary heart disease. N Engl J Med.

[18] Healy B (1991). The Yentl syndrome. N Engl J Med.

[19] Steingart RM, Packer M, Hamm P, Coglianese ME, Gersh B, Geltman EM, Sollano J, Katz S, Moye L, Basta LL (1991). Sex differences in the management of coronary artery disease. Survival and ventricular enlargement investigators. N Engl J Med.

[20] Westergaard D, Moseley P, Sørup F, Baldi P, Brunak S (2019). Population-wide analysis of differences in disease progression patterns in men and women. Nat Commun.

[21] Gagliardi AR, Dunn S, Foster A, Grace SL, Green CR, Khanlou N, Miller FA, Stewart DE, Vigod S, Wright FC (2019). How is patient-centred care addressed in women's health? A theoretical rapid review. BMJ Open.

[22] Gagliardi AR, Green C, Dunn S, Grace SL, Khanlou N, Stewart DE (2019). How do and could clinical guidelines support patient-centred care for women: content analysis of guidelines. PLoS One.

[23] Ramlakhan JU, Foster AM, Grace SL, Green CR, Stewart DE, Gagliardi AR (2019). What constitutes patient-centred care for women: a theoretical rapid review. Int J Equity Health.

[24] Celik H, Lagro-Janssen T, Widdershoven G, Abma T (2011). Bringing gender sensitivity into healthcare practice: a systematic review. Patient Educ Couns.

[25] Arksey H, O'Malley L (2005). Scoping studies: towards a methodological framework. Int J Soc Res Methodol.

[26] Heidari S, Babor TF, De Castro P, Tort S, Curno M (2016). Sex and gender equity in research: rationale for the SAGER guidelines and recommended use. Res Integr Peer Rev.

[27] Al-Khatib SM, Hellkamp AS, Hernandez AF, Fonarow GC, Thomas KL, Al-Khalidi HR, Heidenreich PA, Hammill S, Yancy C, Peterson ED (2012). Trends in use of implantable cardioverter-defibrillator therapy among patients hospitalized for heart failure: have the previously observed sex and racial disparities changed over time?. Circulation.

[28] Asdaghi N, Romano JG, Wang K, Ciliberti-Vargas MA, Koch S, Gardener H, Dong C, Rose DZ, Waddy SP, Robichaux M (2016). Sex disparities in ischemic stroke care: FL-PR CReSD study (Florida-Puerto Rico collaboration to reduce stroke disparities). Stroke.

[29] Batista LE, Rattner D, Kalckmann S, MCGd O (2016). Humanização na atenção à saúde e as desigualdades raciais: uma proposta de intervenção. Saude Soc.

[30] Figueiredo R, Ayres JRCM (2002). Intervenção comunitária e redução da vulnerabilidade de mulheres às DST/ Aids em São Paulo, SP. Rev Saude Publica.

[31] Fine D, Warner L, Salomon S, Johnson DM (2017). Interventions to increase male attendance and testing for sexually transmitted infections at publicly-funded family planning clinics. J Adolesc Health.

[32] Fonarow GC, Abraham WT, Albert NM, Stough WG, Gheorghiade M, Greenberg BH, O'Connor CM, Sun JL, Yancy C, Young JB (2009). Age- and gender-related differences in quality of care and outcomes of patients hospitalized with heart failure (from OPTIMIZE-HF). Am J Cardiol.

[33] Fotso JC, Higgins-Steele A, Mohanty S (2015). Male engagement as a strategy to improve utilization and community-based delivery of maternal, newborn and child health services: evidence from an intervention in Odisha, India. BMC Health Serv Res.

[34] Glickman SW, Granger CB, Ou FS, O'Brien S, Lytle BL, Cairns CB, Mears G, Hoekstra JW, Garvey JL, Peterson ED, Jollis JG (2010). Impact of a statewide ST-segment-elevation myocardial infarction regionalization program on treatment times for women, minorities, and the elderly. Circ Cardiovasc Qual Outcomes.

[35] Haider A, Adler RR, Schneider E, Uribe Leitz T, Ranjit A, Ta C, Levine A, Harfouch O, Pelaez D, Kodadek L (2018). Assessment of patient-centered approaches to collect sexual orientation and gender identity information in the emergency department: the EQUALITY study. JAMA Netw Open.

[36] Hinohara TT, Al-Khalidi HR, Fordyce CB, Gu X, Sherwood MW, Roettig ML, Corbett CC, Monk L, Tamis-Holland JE, Berger PB (2017). Impact of regional Systems of Care on disparities in care among female and black patients presenting with ST-segment-elevation myocardial infarction. J Am Heart Assoc.

[37] Huded CP, Johnson M, Kravitz K, Menon V, Abdallah M, Gullett TC, Hantz S, Ellis SG, Podolsky SR, Meldon SW (2018). 4-step protocol for disparities in STEMI care and outcomes in women. J Am Coll Cardiol.

[38] Jani SM, Montoye C, Mehta R, Riba AL, DeFranco AC, Parrish R, Skorcz S, Baker PL, Faul J, Chen B (2006). Sex differences in the application of evidence-based therapies for the treatment of acute myocardial infarction: the American College of Cardiology's guidelines applied in practice projects in Michigan. Arch Intern Med.

[39] Laisser RM, Nystrom L, Lindmark G, Lugina HI, Emmelin M (2011). Screening of women for intimate partner violence: a pilot intervention at an outpatient department in Tanzania. Glob Health Action.

[40] Lau BD, Haider AH, Streiff MB, Lehmann CU, Kraus PS, Hobson DB, Kraenzlin FS, Zeidan AM, Pronovost PJ, Haut ER (2015). Eliminating health care disparities with mandatory clinical decision support: the venous thromboembolism (VTE) example. Med Care.

[41] Lewis WR, Ellrodt AG, Peterson E, Hernandez AF, LaBresh KA, Cannon CP, Pan W, Fonarow GC (2009). Trends in the use of evidence-based treatments for coronary artery disease among women and the elderly: findings from the get with the guidelines quality-improvement program. Circ Cardiovasc Qual Outcomes.

[42] Low TT, Chan SP, Wai SH, Ang Z, Kyu K, Lee KY, Ching A, Comer S, Tan NQP, Thong E (2018). The women's heart health programme: a pilot trial of sex-specific cardiovascular management. BMC Womens Health.

[43] Mehta RH, Bufalino VJ, Pan W, Hernandez AF, Cannon CP, Fonarow GC, Peterson ED (2008). Achieving rapid reperfusion with primary percutaneous coronary intervention remains a challenge: insights from American Heart Association's get with the guidelines program. Am Heart J.

[44] Sehgal AR (2003). Impact of quality improvement efforts on race and sex disparities in hemodialysis. JAMA.

[45] Walsh MN, Yancy CW, Albert NM, Curtis AB, Gheorghiade M, Heywood JT, Inge PJ, McBride ML, Mehra MR, O’connor CM (2010). Equitable improvement for women and men in the use of guideline-recommended therapies for heart failure: findings from IMPROVE HF. J Card Fail.

[46] Wei J, Mehta PK, Grey E, Garberich RF, Hauser R, Bairey Merz CN, Henry TD (2017). Sex-based differences in quality of care and outcomes in a health system using a standardized STEMI protocol. Am Heart J.

[47] White RO, DeWalt DA, Malone RM, Osborn CY, Pignone MP, Rothman RL (2010). Leveling the field: addressing health disparities through diabetes disease management. Am J Manag Care.

[48] Williams EC, Lapham GT, Rubinsky AD, Chavez LJ, Berger D, Bradley KA (2017). Influence of a targeted performance measure for brief intervention on gender differences in receipt of brief intervention among patients with unhealthy alcohol use in the veterans health administration. J Subst Abus Treat.

[49] Smaha LA (2004). The American Heart Association get with the guidelines program. Am Heart J.

[50] Dielissen P, Verdonk P, Waard MW, Bottema B, Lagro-Janssen T (2014). The effect of gender medicine education in GP training: a prospective cohort study. Perspect Med Educ.

[51] Phillips SP (2008). Measuring the health effects of gender. J Epidemiol Community Health.

