# Supplementary material for: Gender disparities in clinical practice: are there any solutions? Scoping review of interventions to overcome or reduce gender bias in clinical practice
Source: Int J Equity Health. 2020 Sep 22;19:166. doi: 10.1186/s12939-020-01283-4 (PMC7510055; doi:10.1186/s12939-020-01283-4)
Supplement: Supplementary file 1 — Additional file 1. Appendix 1. [file 12939_2020_1283_MOESM1_ESM.docx]

| **Reference** | **health issue/ clinical setting/evaluation** | **Intervention** | **Outcome** |  |
| --- | --- | --- | --- | --- |
| **Cardiovascular Health** | | | | |
| Huded et al,  2018  USA | ST-Elevation Myocardial infarction  Hospital  Emergency department  Evaluation with pre and post comparison and routine data | Four-step STEMI protocol:  1) implementing emergency department physician cardiac catheterization lab activation criteria without requiring delay for consultation with cardiology. 2) Standardized early triage and management of STEMI patients including the administration of guideline-directed medical therapy by using a STEMI Safe Handoff Checklist. 3) Policy of immediate transfer to an immediately available cardiac catheterization lab at all times to avoid patient delays in awaiting readiness of the cardiac catheterization lab. 4) Transition to a radial first approach for vascular access in primary PCI among suitable patients. | In the comprehensive four-step STEMI protocol, gender disparities in GDMT (84% vs. 80%, P=0.320), D2BT (89min [68, 106] vs. 91min [68, 114], P=0.150), and in-hospital adverse events resolved. The absolute gender difference in 30-day mortality decreased from the control group (6.1% higher in women, P=0.002) to the comprehensive four-step STEMI protocol (3.2% higher in women, P=0.090). |  |
| Low et al,  2018  Singapore | Any cardiovascular condition that required regular follow-up  Cardiology clinics  Evaluation with randomised control group and non-routine quantitative data | Sex-tailored Women’s Heart Health Programme (management by an all-women multidisciplinary team and received culture-centred health intervention workshops, designed through in-depth interviews with the participants) | Patients in intervention group had better control of diabetes mellitus (lower HbA1c of 0.63% [CI 0.21-1.04], p = 0.015) and lower body-mass-index (0.74 kg/m2 [CI 0.02-1.46], p = 0.092) at 1 year, but there was no significant difference in blood pressure or lipid control. Overall, there was a trend towards better risk factor control, 31.6% of intervention group versus 26.5% of control group achieved improvement in at least 1 CV risk factor control to target range. There was no significant difference in incidence of cardiovascular events, quality of life, or domains in knowledge, attitudes, intention and practices. |  |
| Hinohara et al,  2017  USA | ST-Elevation Myocardial infarction  Hospital  Evaluation with pre and post comparison and routine data | The STEMI Systems Accelerator project was a nationwide effort to organize and implement STEMI reperfusion therapy in 16 regions across the United States and included 484 hospitals and 1253 EMS agencies. The enrollment criteria for each region included the following: (1) 70% of PCI-capable hospitals in the region were participating in the National Cardiovascular Data Registry’s ACTION Registry–Get With The Guidelines (AR-G) program; (2) there was defined organization of regional leadership; (3) common protocols were established for the diagnosis and treatment of patients with STEMI presenting to EMS personnel or hospitals lacking PCI facilities; (4) there was agreement to enter patients into the AR-G for 6 consecutive quarters; and (5) regional leadership participated in a 2-day national training session directed by study faculty reviewing current evidence, guidelines, and approaches to regional STEMI care. | Change in the proportion of patients treated within guideline goals was compared between sex and race subgroups for patients presenting directly to hospitals capable of performing percutaneous coronary intervention (n=18 267) and patients requiring transfer (n=5542). The intervention was associated with an increase in the proportion of men treated within guideline goals that presented directly (58.7–62.1%, P=0.01) or were transferred (43.3–50.7%, P<0.01). An increase was also seen  among white patients who presented directly (57.7–59.9%, P=0.02) or were transferred (43.9–48.8%, P<0.01). There was no change in the proportion of female or black patients treated within guideline goals, including both those presenting directly and transferred. |  |
| Wei et al,  2017  USA | ST-Elevation Myocardial infarction  Hospital  Community hospitals and clinics  Evaluation without comparison group, routine data. | Standardized STEMI protocol and an organized transfer system (Standardized protocols included a transfer plan (ambulance or helicopter). | Pre-revascularization medications and PCI were performed with same frequencies, but womenwere  less likely to receive statin or antiplatelet therapy at discharge. After age adjustment, women had similar in-hospital mortality to men (5.1% vs 4.8%, P = .60) despite slightly longer door-to-balloon time (95 vs 92 minutes, P = .004). Five-year follow-up confirmed absence of a sex disparity in age-adjusted survival post-STEMI. |  |
| Asdaghi et al, 2016  USA | Stroke  Hospitals  Evaluation without comparison group, routine data. | Get with the Guidelines-Stroke. GWTG initiative focused on the redesign of hospital systems of care to improve the quality of care of patients with coronary artery disease. The GWTG is based on a collaborative model and the Internet-based Patient Management includes interactive learning sessions, teleconference, and electronic communication between multidisciplinary teams from hospitals in a variety of settings to facilitate the transfer of the “how-to” necessary to produce system-wide change. | As compared to men, women were older (73±15 vs. 69±14 years; p<0.0001), more hypertensive (67% vs. 63%, P<0.0001) and had more atrial fibrillation (19% vs. 16%; p<0.0001). DFC was slightly lower in women than men (OR 0.96, 95% CI 0.93–1.00). Temporal trends in DFC improved substantially and similarly for men and women, with a 29% absolute improvement in women (p for trend <0.0001) and 28% in men (p for trend <0.0001), p value for time-by-sex interaction of 0.13. Women were less likely to receive thrombolysis (OR 0.92, 95%CI 0.86–0.99, P=0.02) and less likely to have a door to needle time (DTN) <1 hour (OR 0.83, 95%CI, 0.71–0.97, p=0.02) as compared to men |  |
| Lau et al,  2015  USA | Trauma patients with Venous Thromboembolism  Hospital  Evaluation with pre and post comparison and routine data | A mandatory, service-specific Computerized clinical decision support tool to improve prescription of best-practice VTE prophylaxis for all hospitalized patients | Before implementation, the proportion of male trauma patients prescribed risk-appropriate VTE prophylaxis was significantly higher (69.5% vs. 55.1%, p=0.045). After implementation, compliance increased significantly for both male (85.7%) and female (81.2%) patients and there were no differences between groups (p=0.078) |  |
| Al-Khatib et al 2012  USA | Heart failure  Hospital  Evaluation without comparison group, routine data. | Get With The Guideline- Health Failure  GWTG initiative focused on the redesign of hospital systems of care to improve the quality of care of patients with coronary artery disease. The GWTG is based on a collaborative model and the Internet-based Patient Management includes interactive learning sessions, teleconference, and electronic communication between multidisciplinary teams from hospitals in a variety of settings to facilitate the transfer of the “how-to” necessary to produce system-wide change. | After adjustment for potential confounders, ICD use increased significantly in the overall study population during 2005 to 2007 (odds ratio, 1.28; 95% confidence interval, 1.11–1.48 per year; P 0.0008) and in black women (odds ratio, 1.82; 95% confidence interval, 1.28 –2.58 per year; P 0.0008), white women (odds ratio, 1.30; 95% confidence interval, 1.06 –1.59 per year; P 0.010), black men (odds ratio, 1.54; 95% confidence interval, 1.19 –1.99 per year; P .0009), and white men (odds ratio, 1.25; 95% confidence interval, 1.06 –1.48 per year; P 0.0072). The increase in ICD use was greatest among blacks. |  |
| Walsh et al  2010  USA | Heart failure  Cardiology practices  Evaluation with pre and post comparison and routine data | Intervention components included a guideline-based clinical decision support tool kit, educational materials, practice specific data reports, benchmarked quality-of-care reports, and structured educational and collaborative opportunities. The intervention also included evidence-based best practices algorithms, clinical pathways, standardized encounter forms, checklists, pocket cards, chart stickers, and patient education and other materials. | At baseline, women were less likely than men to be treated with anticoagulation and ICD. Significant improvements in 6 of 7 quality measures were evident at 24 months for both sexes. The absolute magnitude of improvement was similar for 5 measures and significantly better in women for CRT, ICD, and composite care. |  |
| Glickman et al, 2010  USA | ST-Elevation Myocardial infarction  Hospital  Evaluation with pre and post comparison and routine data | The RACE program involved 65 hospitals, including 10 PCI and 55 non-PCI hospitals. RACE focused on the coordination of each aspect of care from the initial emergency medical response to reperfusion itself, that is fibrinolytic therapy or primary PCI whichever was most appropriate for a given setting. he RACE project was created by an alliance between national and regional professional societies, a local payer, the pharmaceutical, and healthcare providers, including emergency medical services, emergency medicine, cardiology, and hospital administrations. The main outcomes of interest are rates of reperfusion and time to treatment. | There was a reduction in baseline  treatment disparities in door-to-ECG times in women versus men (4.4-minute reduction in difference; 95% CI, 8.1 to  0.4; P 0.03). After Reperfusion of Acute Myocardial Infarction in North Carolina Emergency Departments, an  age–treatment time gap persisted in the elderly, relative to younger patients. |  |
| Fonarow et al, 2009  USA | Heart Failure  Hospital  Evaluation without comparison group, routine data | The OPTIMIZE-HF Process-of-Care Improvement Program provided participating hospitals with materials for improving treatment and discharge plans for optimal patient management and included evidence-based best practice algorithms (detailed algorithms on the indications, contraindications,  dosing, and monitoring steps for each evidence based HF therapy), along with comprehensive patient education materials and resources. These tools were based on published HF guidelines from the American College of  Cardiology, the American Heart Association, and the Heart Failure Society of America | Appropriate angiotensin-converting enzyme inhibitor/angiotensin receptor blocker and -blocker use were similar between women and men (p 0.244 and p 0.237, respectively). However, compared with men, fewer women received hospital discharge instructions (p <0.001) and the length of stay was longer (p <0.001). |  |
| Lewis et al, 2009  USA | Acute myocardial infarction  Hospitals  Evaluation without comparison group, routine data | Get with the Guidelines-AMI  GWTG initiative focused on the redesign of hospital systems of care to improve the quality of care of patients with coronary artery disease. The GWTG is based on a collaborative model and the Internet-based Patient Management includes interactive learning sessions, teleconference, and electronic communication between multidisciplinary teams from hospitals in a variety of settings to facilitate the transfer of the “how-to” necessary to produce system-wide change. | This study demonstrated that participating in the AHA GWTG-CAD program, a quality-improvement program, was associated with increased guideline adherence over time irrespective of sex or age for patients hospitalized with CAD. |  |
| Mehta et al 2008  USA | ST-Elevation Myocardial infarction  Hospitals  Evaluation without comparison group, routine data | Get with the guidelines-AMI. GWTG initiative focused on the redesign of hospital systems of care to improve the quality of care of patients with coronary artery disease. The GWTG is based on a collaborative model and the Internet-based Patient Management includes interactive learning sessions, teleconference, and electronic communication between multidisciplinary teams from hospitals in a variety of settings to facilitate the transfer of the “how-to” necessary to produce system-wide change. | Relative to their peers, patients aged ≥65 years (103 [IQR 74-153] vs 93 [IQR 67-133] minutes), women (103 [IQR 73-154] vs 94 [IQR 68-135] minutes), and minorities (108 [IQR 77-162] vs 95 [IQR 68-136] minutes) had significantly longer median D2B times. These subgroup disparities in the D2B persisted over the study period as compared with their peers |  |
| Jani et al  2006  USA | Acute Myocardio infarction patients, physicians,  and nurses  Evaluation with pre and post comparison and routine data | The GAP project fosters systems-  based care from admission to discharge by standard orders,  and a discharge tool to improve evidence-based indicator  rates and long-term mortality in patients with AMI in Michigan, this study compared the success of GAP in men vs women. | Use of evidence-based care, including use of beta-blockers and aspirin in men and women at hospital discharge was higher in the post-GAP sample (P .01 for all). Use of the discharge tool promoted by the GAP program was independently protective against death at 1 year in women (adjusted odds ratio, 0.46; 95% confidence interval, 0.27-0.79), and a trend existed for similar results in men (adjusted odds ratio, 0.62; 95% confidence interval, 0.36-1.06). However, the tool was used slightly less often with women (27.9% vs 33.96%; P=.003). |  |
| **Sexual and reproductive health** | | | | |
| Fine et al,  2017  USA | Sexually Transmitted Infections Family Health Centers  Evaluation with a non-randomised group, non-routine mixed data | 1) In-reach: Clinic staff members were trained on using in-reach strategies with their female clients by encouraging women to inform male partners, friends, and relatives about reproductive health services. 2) Outreach: Clinic staff made presentations to community-based organizations and local health, social service, and correctional agencies about available male reproductive health services, 3) Clinic efficiency: Patient flow analyses were implemented to help program managers identify and resolve service bottlenecks for clients transitioning between clinic stations and to reduce wait times. Intervention sites did not receive additional resources to increase staff or program hours. 4) Staff training: Staff members were provided training on the “culture of men” and providing services to male clients. The training included gender differences in communication and decision-making, influences of socialization on male sexual health, and the possible impact of male stereotyping on services. Staff reviewed clinic visit components, including determining service needs, contraceptive options, medical history, sexual health assessment, sexually transmitted disease services, preventive health services, and risk counseling. Clinical staff also received skill-based training on conducting male genital exams, including documentation of normal growth and development and other common genital findings.  5) Clinic environment: Staff assessed intervention sites’ physical settings to identify possible areas for improvements, for example, incorporating male-appropriate brochures and materials in waiting rooms and medical posters in exam rooms. Clinic intake forms, policies, and protocols were updated to better reflect male clients and their health care needs. | From preintervention to postintervention, intervention clinics significantly increased the number of male visits (4,004 to 8,385; D ¼ þ109%); for comparison clinics, male visits increased modestly (3,822 to 4,500; D ¼ þ18%). The proportion of male clinic visits where chlamydia testing was performed increased in intervention clinics (35% to 42%; p < .001) but decreased in comparison clinics (37% to 33%; p < .001).  Multilevel interventions designed to increase male client volume and sexually  transmitted infection testing services in family planning clinics succeeded without affecting female  client volume or services. |  |
| Haider et al, 2017  USA | Sexual orientation and gender identity  Emergency Department  Evaluation with a non-randomised group, non-routine mixed data | A multidisciplinary stakeholder advisory board comprised of patients, physicians, and LGBT health advocates helped to inform our study design. Based on results from phase 1 of the EQUALITY Study, the SAB chose the 2 most viable and preferred methods of SOGI collection, as well as relevant outcome measures to use in the interventional study. Two Sexual orientation and gender identity collection approaches. Both modes, nurses and registrars received several education sessions by experts and LGBT patient advocates to explain the need for routine SOGI information collection. We also incentivized staff with gift cards for those who collected this information on the most patients.  ED nurses were requested to collect SOGI information from their patients as part of the social history portion of the patient assessment and enter it directly into the EHR, which had been previously modified to collect this data"registrars asked patients to confidentially complete a demographics information form that included SOGI information,  administered via iPad at the 2 sites in Boston, Massachusetts, or on paper at the sites in Baltimore, Maryland. Electronic forms were automatically attached to the patient’s EHR, while paper forms were immediately entered into the EHR by staff. In both modes, researchers ran analytic reports every hour (when on duty from 7 AM-10 PM) to identify patients in the ED who were eligible for a survey to ascertain which method of SOGI was preferable to them" | Sexual or gender minority patients had significantly better Communication Climate Assessment Toolkit scores with nonverbal registrar form collection compared with nurse verbal collection (mean [SD], 95.6 [11.9] vs 89.5 [20.5]; P = .03). No significant differences between the 2 approaches were found among non-SGM patients |  |
| Batista et al, 2016  Brazil | Maternal mortality among black women  Hospital  Evaluation without comparison group, non-routine mixed data | The project’s seven stages were: sensitization and negotiation within the Secretaria de Estado da Saúde de São Paulo (SES-SP) [State Secretariat of Health of São Paulo]; sensitization and negotiation with the Brazilian Ministry of Health; sensitization and establishment of an agreement with the health service; diagnosis of the situation in the hospital; sensitizing the professionals towards gender and race issues, introducing the race data collection in the forms of the hospital’s data bank; training hospital staff; forming social movements within the neighborhood | The project’s results were: the collection of data on race became routine, and prompted the Ministry of Health to adopt this information request in all its forms; professionals were trained in women’s health care and sensitized to the specificities of the black population; and there were changes in how the health care team perceives the presence of the father at birth, creating the campaign “Fathers are not visitors”. The project was effective in broaching those complex issues and it may be replicated elsewhere. |  |
| Fotso et al, 2015  India | Maternal and child health  Community health center or district hospital in the few and health activists  Evaluation without comparison group, non-routine qualitative data | Training male Community Health Workers (CHWs) known as Male Health Activists (MHAs) to complement the work of Accredited Social Health Activists (ASHAs) and target outreach to men. | Participants’ responses are broadly organized around the facilitation of ASHAs’ work by MHAs, and male engagement activities undertaken by MHAs. More specifically, the narratives reflected gender-based divisions of work and space in three core areas of delivery and use of MNCH services: escorting women to health centres for facility-based deliveries; mobilizing women and children to attend Village Health and Nutrition Days and  Immunization Days; and raising awareness among men on MNCH and family planning. |  |
| Figueiredo et al, 2002  Brazil | Sexually Transmitted Infections and HIV among women  Outpatient clinic  Evaluation with pre and post comparison and non-routine qualitative data | The following actions were put in place: training of health professionals from the local outpatient clinic, availability of prevention resources (male and female condoms), educational groups, educational materials and community radio programs. | We highlight the increase in the demand for condoms and the interest in female condoms; relevant differences related to gender and age and adherence to the proposed activities; and good results in raising awareness and training of health professionals, although with limits in maintaining involvement with preventive activities. |  |
| **Gender Violence** | | | | |
| Laisser et al, 2011  Tanzania | Gender based violence  Outpatient department of a hospital  Evaluation without comparison group and non-routine mixed data | Prior to screening, 39 health care workers attended training on gender-based violence and the  suggested screening procedures. Seven health care workers were arranged to implement screening in 3 weeks,  during March April 2010. For screening evaluation, health care workers were observed for their interaction  with clients. | Of the 102 women screened, 78% had experienced emotional, physical, or sexual violence. Among them, 62% had experienced IPV, while 22% were subjected to violence by a relative, and 9.2% by a work mate. Two-thirds (64%) had been abused more than once; 14% several times. Almost one-quarter (23%) had experienced sexual violence. Six of the health care workers interacted well with clients but three had difficulties to follow counselling guidelines. FGDs and narratives generated three categories Just asking feels good implied a blessing of the tool; what next? indicated ethical dilemmas; and fear of becoming a ‘women’ hospital only indicated a concern that abused men would be neglected. |  |
| **Other** | | | | |
| Williams et al,  2017  USA | Unhealthy drinking  veterans medical centre  Evaluation with pre and post comparison group and non-routine quantitative data | Brief intervention both before and after implementation and evaluate whether gender differences in receipt of brief intervention changed in relation to implementation of brief intervention with a performance measure. | Among patients optimally eligible for BI (n=51,272, 8,206 women and 43,066 men), the prevalence of BI increased more steeply for men than women after implementation (interaction p-value <0.0001). Pre-implementation rates of BI were 21% (95% CI, 18-24) for women and 26% (95% CI, 24-29) for men, and post-implementation rates were 32% (95% CI, 30-34) for women and 47% (95% CI, 45-49) for men. |  |
| White et al, 2011  USA | Diabetes  Primary health care centre  Evaluation with a randomised control group, with non-routine quantitative data | Supplementary support program (intensive management from clinical pharmacists and from a diabetes care coordinator who provided diabetes education, applied algorithms for treating glucose level abnormalities and decreasing cardiovascular risk, used an electronic registry to proactively identify patients in need of additional care, and addressed barriers to care. Treatment algorithms provided guidance on when to contact patients and information about titration or addition of new medications for glucose control and cardiovascular risk reduction.) | One hundred ninety-three of 217 enrolled patients (88.9%) had complete 12-month follow-up data. Patients in the intervention group had significantly greater improvement in A1C level than the control group (−2.1% vs −1.2%, P = .007). In multivariate analysis, no significant differences were observed in improvement in A1C level when stratified by age, race/ethnicity, income, or insurance status, and no interaction effect was observed between any covariate and intervention status. Among intervention patients, we observed similar labor inputs regardless of age, race/ethnicity, sex, education, or whether goal A1C level was achieved. |  |
| Sehgal et al 2003  USA | Renal failure  Centres for medicare and medicaid services  Evaluation without comparison group, with routine data | Medicare-funded quality improvement project involving monitoring of patient outcomes, feedback of performance data, and education of clinicians at dialysis centres. | The gap between female and male patients decreased from 23% to 9% over the same period (P = .008). The proportion of all patients with adequate hemoglobin levels increased 3-fold. The proportion of all patients with adequate albumin levels remained unchanged. Race and sex disparities in anemia management and nutritional status did not change significantly. |  |

STEMI: ST elevation myocardial infarction, D2BT: Door to bed time, GWTG: Get with the Guidelines, ICD: implantable cardioverter defibrillator
